# Prevalence, associations and sex-related disparities of hypertension in South Asians: Evidence from a national survey of Sri Lanka

**DOI:** 10.1371/journal.pone.0361894

**Published:** 2026-11-02

**Authors:** Chamila Mettananda, Ravini Premaratna, Randula Mallawa, Upeksha Fernando, Arundika Senaratne, Udara Perera, Champika Wickramasinghe

**Affiliations:** 1 Department of Pharmacology, Faculty of Medicine, University of Kelaniya, Colombo, Sri Lanka; 2 NCD Directorate, Ministry of Health, Colombo, Sri Lanka; Washington University in St Louis, Bursky School of Public Health, UNITED STATES OF AMERICA

## Abstract

**Background:**

Blood pressure(BP) control remains suboptimal worldwide. Sex-related disparities in hypertension have been reported, but data from South Asia are limited. Understanding the epidemiology and sex-related differences is essential to improve BP control.

**Objectives:**

To assess the prevalence, associations and sex-related disparities of hypertension among Sri Lankans.

**Methods:**

We analysed secondary data from the Sri Lanka STEPS Survey 2021, a nationally representative, community-based, cross-sectional study. Hypertension was defined as BP ≥ 140/90 mmHg or current antihypertensive use; controlled BP as BP < 140/90 mmHg. Prevalences, associations, and sex-related disparities in hypertension, undiagnosed hypertension, and BP control were assessed using logistic regression.

**Results:**

Data from 4,634 adults(mean age 43.5(weighted CI 43.0–44.1) years; female 54.8 (53.1–56.5)%) were analysed. Overall, 37.2(35.5–38.9)% had hypertension, of whom 60.0(58.1–63.4) % were previously undiagnosed. Among those previously diagnosed, 73.6 (73.6, 69.6–77.2)% were on treatment, but only 38.0(33.0–44.20)% achieved BP targets. The rates of hypertension prevalence (p = 0.600), treated hypertension (p = 0.398), and controlled blood pressure (p = 0.819) in females and males did not differ significantly by sex. However, undiagnosed hypertension was significantly higher in males (69.4%, 65.3–73.1) than in females (53.6%, 50.1–57.0) (p < 0.001). Differences in the associations of hypertension by sex were noted; diabetes was associated only in females (p < 0.001), while wealth quintile (p < 0.001) and 24-hour urinary salt excretion (p = 0.011) were associated only in males. Overweight/obesity was shown to have a significant association with undiagnosed hypertension only in males (p = 0.017) in stratified analyses.

**Conclusions:**

More than half of the hypertensives in this South Asian population were previously undiagnosed. Of hypertensives, ¾ were treated, but only 1/3 achieved treatment goals. Prevalence, treatment and control rates of hypertension were not different between females and males, but undiagnosed hypertension was more common in men. Therefore, the emphasis should be on improving the detection of hypertension, especially in men, and on treating to targets in both sexes.

## Introduction

Cardiovascular diseases (CVDs) are the leading cause of mortality and morbidity worldwide, including Asia and also in Sri Lanka [1–3]. Elevated blood pressure (BP), the so-called hypertension, is the most important modifiable risk factor for almost all CVDs [4,5]. Click or tap here to enter text. Despite being a modifiable risk factor, BP goal achievement (i.e., controlled BP) is suboptimal, with only around 50% in high-income countries and 20% in low- and middle-income countries (LMIC) achieving BP control [6]. Previously reported studies covering only a few districts reported a prevalence of hypertension of 23% in 2005 and 33% in 2021 in Sri Lanka [7,8]. A nationwide study conducted in 2018−19 reports that only 20% of individuals have controlled hypertension, which is far below the level expected given high health literacy and free health services [9]. Therefore, more recent, nationally representative data, including associations with poor blood pressure control, are needed to plan and implement primary and secondary preventive measures for hypertension to reduce the burden of hypertension in Sri Lanka.

Identifying associations with poor blood pressure control and addressing them is important to achieve a country's blood pressure control targets. Sex-related disparities in hypertension have been observed, and targeting those helps to improve blood pressure control efficiently [10,11]. A considerable sex difference in the prevalence, awareness, treatment and control of hypertension is reported from India, China and Sweden [12–14]. Significantly fewer cardiovascular investigations, such as echocardiography, exercise stress testing, coronary angiogram and sleep apnoea test, were done in women than in men [15]. However, data on sex- related differences in the epidemiology and associations of hypertension are lacking from most South Asian countries. Identifying sex-related disparities helps implement targeted interventions to improve primordial prevention of hypertension and primary and secondary prevention of CVDs across the entire South Asian region.

Therefore, we aimed to study the prevalence, control, and associations of hypertension among Sri Lankans, by sex, and to examine sex-related disparities in blood pressure control using national data from Sri Lanka to guide LMICs in South Asia.

## Methodology

We analysed secondary data from the World Health Organisation's (WHO) STEPwise approach to noncommunicable disease (NCD) risk factor surveillance (STEPS) Survey – Sri Lanka 2021. The methodology for this study is reported elsewhere [16]. The research team accessed de-identified data for this study on 25 February 2025. The authors did not have access to information that could identify individual participants during or after data collection.

A total of 6267 nationally representative adults aged 18–69 who had lived in their current residence for more than 6 months at the time of sampling were studied using multistage stratified cluster sampling in 2021. The sample was stratified by district and area (urban, rural, and estate areas). A primary sampling unit (PSU) was considered a cluster. The framework of PSUs was based on the census blocks prepared for the 2011 Census of Population and Housing, which were updated in 2018. Six hundred forty-four PSUs were selected using the Probability Proportionate Sampling (PPS) method, based on the population of the area (10). Individuals mentally or physically unfit to be included in the study were excluded from the survey. A complete case analysis was performed on the selected patients. Data was collected in three steps: Step 1 – an interviewer administered a questionnaire on sociodemographic and behavioural risk factors for NCDs, Step 2 physical measurements, e.g., blood pressure, height, weight, waist circumference and spot urine collection, Step 3 – biochemical measurements such as the fasting plasma glucose and serum total cholesterol levels. The overall response rate was 81% [16].. Participants were not paid for participation in the study.

Details of the data collection have been previously published [16]. The level of education was determined by the total number of completed years of schooling (excluding preschool). Participants were grouped into six mutually exclusive categories based on the highest qualification attained: (1) no formal schooling, (2) up to Grade 5, (3) passed Grades 6–10, (4) passed G.C.E. ordinary level (O/L), (5) passed G.C.E. advanced level (A/L), (6) degree or higher level of education. The main occupation categories were defined according to national STEPS coding as: “engaged in economic activity” (any paid employment or self-employment), “seeking and available for work” (unemployed and actively seeking work), “student,” “engaged in household activities” (full-time unpaid domestic work/caregiving), “retired”, “unable to work (elderly/disabled)”, and “other”. The wealth index was calculated using *the formula published online at* *http://www.health.gov.lk/moh_final/english/others.php?pid=127, and* patients were categorised into five wealth quintiles (from 1 (poorest) to 5 (richest)) [16].

Blood pressure was measured by trained field staff, who consisted of medical and nursing graduates, using standardised digital automatic blood pressure meters (Bosch & Sohn Medicus, Uno, or OMRON M6) with appropriate universal cuffs. Three blood pressure measurements were obtained 3 minutes apart, with the first reading taken after a 15-minute resting period. Body mass index (BMI) was calculated from weight and height, and Asian BMI cutoffs (BMI ≥ 23 kg/m^2^) were used to define overweight/obesity. Urinary sodium excretion (mmol/L) was measured in spot urine samples, and the 24-hour urinary sodium excretion was calculated using the Kawasaki equation [17].

The Sri Lanka STEPS 2021 survey was a nationally representative, population-based cross-sectional survey of adults aged 18–69 years, using a multistage stratified cluster sampling design, stratified by district and sector (urban, rural and estate), with census blocks selected as primary sampling units using probability proportional to size sampling. A design effect of 1.6 was used for the main prevalence/sample-size estimation to account for the complex sampling design. Data were collected in three rounds due to the COVID-19 pandemic. 1^st^ round: April 2021 2^nd^ round: November 2021 and 3^rd^ round December 2021. Blood pressure was measured at step 2 of the survey. The survey data collection was completed within three months. Base weights were calculated at the block level. Adjustments for household non-response were made. Population adjustment for STEP1, STEP2, and STEP3 was performed according to WHO recommendations [16].

We extracted de-identified data and selected participants who had complete data on blood pressure and other metabolic risk factors for analysis. Missing data were not imputed. Respondents were asked whether they had been treated for raised blood pressure with medication prescribed by a doctor or other health worker during the past two weeks, and those who said yes were labelled as being treated for hypertension. Hypertension was defined according to the National Guideline for the Management of Hypertension in Secondary and Tertiary Health Care settings, 2021, as having a systolic blood pressure (SBP) ≥ 140 mmHg or a diastolic blood pressure ≥ 90 mmHg, or currently being on treatment with antihypertensives with a prior history of hypertension [18]. The mean of the last two BP readings was taken as the current BP. The BP goal was defined as <140/90 mmHg in individuals already on antihypertensive treatment, according to the National Guideline [18]. For the calculation of hypertension prevalence, the final STEP 2 survey weight was applied; this incorporates the base sampling weight, adjustment for household non-response, and the STEP 2 population adjustment. The rates of hypertension prevalence, treatment uptake, and BP goal achievement were calculated in the total population and stratified by sex using weighted data. They were compared between females and males and across urban, rural, and estate populations.

Statistical analysis was conducted using IBM® SPSS® Statistics 30.0. Continuous variables were assessed for normality and reported as means with standard deviations (SD) if normally distributed, or as medians with interquartile ranges (IQR) if not normally distributed. Differences in continuous variables between groups were compared using the independent-samples t-test (when normally distributed) or the Mann–Whitney U test (when non-normally distributed). Categorical variables were reported as frequencies and percentages, and group comparisons were performed using the chi-square (χ2) test. All assessments were conducted using SPSS's complex samples analysis wizard on weighted data. Disparities in hypertension prevalence, diagnosis, and control between females and males were studied using the chi-square (χ2) test. Factors associated with hypertension in the population, undiagnosed hypertension among patients with hypertension, and uncontrolled blood pressure among treated patients were studied using complex samples logistic regression adjusted for age, sex, level of education, wealth quintiles, cardiovascular risk, diabetes, hyperlipidaemia, obesity, smoking, waist circumference and 24-hour urinary salt excretion in the total sample and stratified by sex. Two-sided statistical tests were reported, and p-values ≤ 0.05 were considered statistically significant.

Ethics approval for the STEPS survey in Sri Lanka was obtained from the Ethics Review Committee of the Sri Lanka Medical Association (ERC/17–021). The secondary use of data was also covered by the same. All procedures were performed in accordance with the principles outlined in the Declaration of Helsinki. All participants provided informed consent prior to their inclusion in the study.

## Results

Data of 4634 participants with BP measurements were analysed. The majority of them, 2914 (54.8%, 53.1–56.5) were female. The baseline characteristics of the study population are presented in Table 1. The mean age of the population was 43.5 (43.0–44.1) years, and males did not differ from females in age (p = 0.497), but did differ in level of education (p = 0.035) and main occupation (p < 0.001). Significantly fewer females were engaged in economic activity (31.2, 29.2–33.3) compared to men (79.5, 77.0–81.8). Males were not different to females in wealth quintiles (0.455) nor in distribution between different sectors of residence (p = 0.129), but differed in marital status (p < 0.001). Males compared to females smoked more (p < 0.001) and had less prevalence of hyperlipidaemia (p < 0.001)., obesity/overweight (p < 0.001)., less abdominal obesity (p < 0.001)., BMI (p < 0.001)., total cholesterol (p < 0.001)., but had more strokes (p = 0.001).

**Table 1:** Characteristics of the study population by sex (weighted analysis).

	Total	Women	Men	P
Sex, n (%, CI)	4634	2914 (54.8%, 53.1-56.5)	1720 (45.2%, 43.5-46.9)	<0.001
Age, mean (CI) years	43.5(43.0-44.1)	43.5(42.8-44.2))	43.6(42.7-44.4)	0.497
Age groups, n (%, CI)				0.580
18-29	667 (22.7, 21.0-24.5))	418(22.2, 20.1-24.5)	249(23.2, 20.7-26.0)	
30-44	1522 (29.1, 27.5-30.7))	1015(30.1, 28.3-32.0)	507(27.9, 25.4-30.5)	
45-59	1481 (30.4, 28.9-32.1)	893(30.1, 28.1-32.2)	588(30.8, 28.5-33.3)	
60-69	964 (167.8, 16.6-19.0)	588(17.6, 16.2-19.2)	376(18.1, 16.2-20.1)	
Marrital status, %(CI)				<0.001
Never married	616(20.4, 18.7-22.2)	296(15.60, 13.7-17.6)	320(26.20, 23.7-29.0)	
Currently married	3452(72.2, 70.5-73.9)	2147(73.20, 71.1-75.3)	1305(71.00, 68.3-73.6)	
Separated	100(1.3, 1.1-1.7)	73(1.70, 1.3-2.3)	27(0.90, 0.6-1.3)	
Divorced	36(0.6, 0.4-0.9)	26(0.80, 0.5-1.3)	10(0.30, 0.2-0.60	
Widowed	356(5.4, 4.8-6.1)	313(8.60, 7.5-9.8)	43(1.50, 1.1-2.2)	
Ethnicity, n(%, CI)				0.467
Sinhala	3228(76.4, 73.6-78.9)	2034(76.7,73.8-79.4)	1194(75.9, 72.6-79.0)	
Sri Lankan Tamil	879(13.2, 11.6-15.0)	544(12.6,10.8-14.6)	335(14.0, 12.0-16.2)	
Indian Tamil	138(2.5, 1.8-3.4)	88(2.5,1.7-3.6)	50(2.5, 1.8-3.7)	
Sri Lankan Moor / Muslim	381(7.7, 6.0-9.9)	241(7.9,6.1-10.3)	140(7.5, 5.6-10.1)	
Malay	2(0.1, 0.0-0.3)	2(0.1, 0.0-0.5)	0(0.0)	
Burgher	2(0.0, 0.0-0.1)	1(0.0, 0.0-0.1)	1(0.0, 0.0-0.2)	
Other	3(0.0, 0.0-0.3)	3(0.1, 0.0-0.5)	0(0.0)	
Refused	1(0.0, 0.0-0.1)	1(0.0, 0.0-01)	0(0.0)	
Level of education, n (%, CI)				0.035
Level 0 – No schooling	142 (2.4, 1.9-3.0)	102(2.8, 0.9-2.4)	40(1.9, 1.3-2.8)	
Level 1 – Up to grade 5	594 (11.3, 10.2-12.6)	350(10.7, 9.4-12.2)	244(12.1, 10.3-14.1)	
Level 2 – Grade 6–10	2108 (43.3, 41.5-45.2)	1285(41.6, 39.3-43.9)	823(45.4, 42.6-48.2)	
Level 3 – Passed G.C.E. O/L	768 (18.4, 17.1-19.8)	502(18.4, 16.8-20.1)	266(18.4, 16.220.8)	
Level 4 – Passed G.C.E. A/L	802 (19.7, 18.2-21.3)	536(21.4, 19.3-23.5)	266(17.6, 15.5-20.1)	
Level 5 – Degree and above	218 (4.8, 4.1-5.8)	138(5.1, 4.1-6.3)	80(4.5, 3.5-5.8)	
Refused to answer	2(0.0, 0.0-0.1)	1(0.0, 0.0-0.1)	1(0.1, 0.0-0.4)	
Main Occupation, n (%, CI)				<0.001
Engaged in economic activity	2316 (53.1, 51.4-54.8)	905(31.2, 29.2-33.3)	1411(79.5, 77.0-81.8)	
Seeking and available work	90 (33.1, 2.5-3.9)	41(2.4, 1.6-3.4)	49(4.0, 3.0-5.4)	
Student	182 (6.9, 5.8-8.1)	110(6.8, 5.6-8.4)	72(6.9, 5.3-8.8)	
Engaged in household activities	1737 (30.6, 29.0-32.3)	1723(55.3, 52.8-57.7)	14(0.7, 0.4-1.4)	
Retired	168 (3.6, 3.1-4.3)	58(1.9, 1.4-2.5)	110(5.7, 4.7-7.0)	
Unable to work (elderly/disabled)	136 (2.6, 2.1-3.1)	75(2.3, 1.7-3.0)	61(2.9, 2.2-4.0)	
Other	5 (0.1, 0.0-0.3)	2(0.1, 0.0-0.6)	3(0.1, 0.0-0.5)	
Wealth index, n (%, CI)				0.455
First (poorest) quintile	928 (14.3, 13.0-15.8)	606(14.8, 13.2-16.4)	322(13.8, 11.9-16.0)	
Second quintile	919 (17.8, 16.6-19.2)	573(17.8, 16.2-19.5	346(17.8, 15.8-20.1)	
Third quintile	963 (21.3, 20.0-22.8)	614(22.3, 20.4-24.3)	349(20.3, 18.2-22.4)	
Fourth quintile	927 (23.0, 21.4-24.6)	580(22.4, 20.5-24.4)	347(2.7, 21.2-26.3)	
Fifth (richest) quintile	897 (23.5, 21.7-25.4)	541(22.7, 20.7-24.9)	356(24.5, 21.8-27.4)	
Sector of residence, n (%, CI)				0.129
Urban	689 (15.8, 13.3-18.6)	417(14.8, 12.3-17.7)	272(17.0, 14.0-20.5)	
Rural	3773 (80.9, 77.8-83.7)	2396(82.2, 79.0-85.0)	1377(79.3, 75.5-82.7)	
Estate	172 (3.3, 2.2-5.0	101(3.0, 2.0-4.6)	71(3.7, 2.3-5.9)	
Cardiovascular risk factors, n (%, CI)				
Current smokers	505 (13.0, 11.7-14.3)	3(0.0, 0.0-0.2)	502(28.6, 26.0-31.4)	<0.001
Hyperlipidaemia	609 (12.3, 11.3-13.5)	409(13.1, 11.7-14.6)	200(11.4, 9.8-13.1)	<0.001
Diabetes mellitus	768 (15.9, 14.7-17.1)	289(15.8, 14.4-17.4)	479(16.4, 14.2-18.0)	0.885
Overweight/obesity*^b^	2733 (57.4, 55.7-59.2)	1870(63.9, 61.7-66.0)	863(49.6, 46.8-52.5)	<0.001
Waist, mean (CI) cm, ^b^	86.1 (85.6-86.7)	86.6(85.9-87.3)	85.6(84.8-86.4)	0.001
BMI, mean (CI) kg/m^2^, ^b^	24.3 (24.1-24.5)	25.1(24.9-25.4)	23.2(22.9-23.5)	<0.001
FBG, mean (CI) mg/dL, ^c^	105 (103.1-106.2)	105(103.3-107.5)	103(101.5-106.1)	0.989
Total cholesterol mg/dL	187(185.5-188.6)	191(189.3-193.4)	182(179.5-184.0)	<0.001
Existing cardiovascular diseases, n (%, CI)				
Stroke	46 (1.0, 0.8-1.4)	21(0.6, 0.4-1.1)	25(1.5, 1.0-2.3)	0.010
Ischaemic heart disease	415 (9.4,8.4-10.5)	238(8.5, 7.3-9.8)	177(10.5, 9.0-12.3)	0.052
Medication History other than antihypertensives. n (%, CI)				
Statin	389 (8.1, 7.3-9.1)	254(8.6, 7.4-9.9)	135(7.6, 6.3-9.0)	0.270
Aspirin	163(3.9, 3.3-4.7)	88(3.4, 2.6-4.3)	75(4.6, 3.7-5.9)	0.061

n number, CI – Confidence intervals, SE – standard error, IQR interquartile range, SBP systolic blood pressure, DBP diastolic blood pressure, FBG fasting blood glucose, BMI body mass index. All values are n (weighted %, CI) unless specified. *on BMI cutoff for Asians; BMI ≥ 23 kg/m^2^. Detailed analysis of the wealth index can be downloaded from (http://www.health.gov.lk/moh_final/english/others.php?pid=127)

The prevalence of hypertension in the population and across different socioeconomic strata is shown in Table 2. In 2021, the population prevalence of hypertension was 37.2% and did not differ by sex (0.600) or by sector of residence (urban, rural, and estate) (p = 0.398). Prevalence of hypertension was significantly reduced with increasing levels of education (p < 0.001) and wealth quintiles (p = 0.001), and was lower among never-married people compared with other marital categories.

**Table 2:** Prevalence of hypertension in the population by sex, sector of residence and socioeconomic strata.

	Weighted prevalence of hypertension N(%, CI)	p-value#
Total study population, n (%, CI)	1826 (37.2, 35.5-38.9)	…
Sex		
Female	1130(36.8, 34.7-38.9)	0.600
Male	696(37.7, 35.1-40.4)	
By the sector of residence, n (%, CI)		
Urban	288 (38.3, 34.1-42.6)	0.398
Rural	1463 (36.8, 34.9-38.6)	
Estate	75 (42.8, 33.7-52.4)	
Marrital status, %(CI)		
Never married	125(14.7, 12.0-17.9)	<0.001
Currently married	1419(41.5, 39.6-43.5)	
Separated	44(47.7, 36.0-59.7)	
Divorced	22(64.65, 45.6-79.8)	
Widowed	206(58.9, 52.5-64.9)	
By the level of education, n (%, CI)		<0.001
No schooling	65 (45.7, 35.6-56.2)	
Up to grade 5	294 (48.3, 43.4-53.4)	
Passed grade 6–10	870 (40.8, 38.4-43.3)	
Passed G.C.E. O/L	285 (34.1, 30.2-38.2)	
Passed G.C.E. A/L	241 (26.6, 23.3-30.2)	
Degree and above	69 (29.0, 22.0-37.1)	
By the Wealth index, n (%, CI)		0.001
Poorest quintile	392 (43.1, 39.1-47.2)	
Second quintile	395 (41.1, 37.3-45.1)	
Third quintile	374 (36.2, 32.8-39.8)	
Fourth quintile	325 (32.9, 29.6-36.4)	
Richest quintile	340 (35.7, 32.1-39.5)	

n number, SE standard error, RR relative risk, CI confidence interval, ^#^Pearson's chi-square test, All values are n (weighted %, CI) unless specified.

Rates of hypertension, prevalence, undiagnosed, treated, and controlled hypertension in the study population and by sex are shown in Table 3. Of the total population, 810 (19.3, 17.8–20.8%) had never checked their blood pressure at least once in their lifetime; significantly more males had never checked their blood pressure than females (P < 0.001). Mean SBP (p < 0.001) and DBP (p < 0.001) were lower in females than in males.

**Table 3:** Prevalence, undiagnosed, treated, and control hypertension in the population by sex.

	Total	Women	Men	P#
Mean SBP (SE) in population, mmHg	128.0 (127.2-128.8)	126(125.3-127.4)	130.0(129.0-131.1)	< 0.001
Mean DBP (SE) in population, mmHg	80.3 (79.8-80.8)	79.0 (78.4-79.6)	81.8 (81.1-82.6)	< 0.001
Had never checked blood pressure before this study, n (%, CI)	810 (19.3, 17.8-20.8)	366(12.8, 11.3-14.5)	444(27.2, 24.7-29.8)	< 0.001
Prevalence of hypertension, n (%, CI)	1826 (37.2, 35.5-38.9)	1130(36.8, 34.7-38.9)	696(37.7, 35.1-40.4)	0.600
Prevalence of undiagnosed hypertension in the population, n (%, CI)	1080 (22.6, 21.2-24.1)	601 (19.7, 18.0-21.5)	479 (26.1, 23.8-28.6)	<0.001
Percentage treated among diagnosed, n (%, CI)	538 (73.6, 69.6-77.2)	376 (72.3, 67.5-76.7)	162 (75.8, 68.7-81.8)	0.398
The percentage controlled among treated, n (%, CI)	208(38.0, 33.5-42.7)	147(38.5, 33.0-44.2)	61 (37.3, 29.4-45.9)	0.819

number, CI confidence interval, SBP systolic blood pressure, DBP diastolic blood pressure. All values are n (weighted %, CI) unless specified. ^#^Pearson's chi-square test

The prevalence of hypertension in the population was 37.2% (CI 35.5–38.9%), and there was no significant difference between females and males (p = 0.600). Prevalence of undiagnosed hypertension in the population was 22.6 (21.2–24.1)%; lower among females (19.7, 18.0–21.5) than males (26.1, 23.8–28.6) (p < 0.001). Among those diagnosed with hypertension, 73.6% (69.6–77.2%) were on treatment; no difference between females and males (p = 0.398). Among those treated for hypertension, 38.0% (33.5–42.7%) had controlled blood pressure; no difference between females and males (p = 0.819).

The factors associated with hypertension in the total population and by sex are presented in Table 4. Increasing age (*P* < 0.001), co-morbid diabetes (P = 0.006), hyperlipidaemia (*P* < 0.001), overweight/obesity (*P* < 0.001) and increasing waist circumference (*P* < 0.001) were positively associated while increasing wealth quintile was negatively (*P* < 0.001) associated with prevalence of hypertension in the total population on logistric regression adjusted for age, sex, level of education, wealth quitiles, presence of diabetes, hyperlipidaemia, overweight/obeseity, current smoking, waist circumference and 24-hour urinary salt excretion in the model of weighted analyses but sex was not significantly associated with the prevalence of hypertension (P = 0.672). Prevalence of hypertension in females was associated with age, presence of diabetes, hyperlipidaemia, overweight/obesity, and waist circumference. Hypertension in males was associated with age, wealth quintiles, hyperlipidaemia, overweight/obesity, waist circumference, and 24-hour urinary salt excretion. Diabetes was associated with hypertension only in females (p < 0.001), whereas wealth quintile (p < 0.001) and 24-hour urinary salt excretion (p = 0.011) were associated only in males.

**Table 4:** Factors associated with the prevalence of hypertension in the population and by sex.

	Total population			Female			Male		
	OR	95% CI	P value*	OR	95% CI	p	OR	95% CI	p
Increasing age	1.06	1.05-1.07	<0.001	1.07	1.06-1.08	<0.001	1.06	1.05-1.07	<0.001
Male sex	1.05	0.83-1.33	0.672	….	….	….	….	….	…
Incresing level of education	1.05	0.96-1.14	0.298	1.00	0.89-1.110	.944	1.14	1.00-1.30	0.71
Increasing wealth quintile	0.86	0.80-0.92	<0.001	0.90	0.82-0.98	0.12	0.82	0.73-0.92	<0.001
Diabetes mellitus	1.35	1.09-1.67	0.006	1.71	1.28-2.78	<0.001	1.03	0.75-1.42	0.847
Hyperlipidaemia	2.41	1.89-3.09	<0.001	2.38	1.75-3.22	<0.001	2.45	1.66-3.61	<0.001
Overweight/obesity**	1.55	1.25-1.91	<0.001	1.51	1.27-2.02	0.006	1.58	1.14-2.18	0.006
Current smoking	1.04	0.80-1.37	0.758	1.84	0.07-49.8	0.701	1.07-	0.82-1.40	0.616
Waist, cm	1.02	1.02-1.03	<0.001	1.03	1.02-1.04	<0.001	1.02	1.01-1.03	0.002
24-hour urinary salt excretion (mmol/L)	1.003	1.000-1.006	0.058	1.00	0.995-1.004	0.885	1.01	1.00-1.01	0.011

*LR Logistic regression, OR odds ratio, CI confidence interval, BMI body mass index, NM never married.* All calculations were done using complex samples – logistic regression. * Logistic regression, adjusted for increasing age, male sex, increasing level of education, wealth quintiles, presence of diabetes mellitus, hyperlipidaemia, overweight/obesity, current smoking, waist circumference and 24-hour urinary salt excretion in the model. ** BMI ≥ 23 kg/m^2^.

Table 5 presents the association of uncontrolled blood pressure among treated patients. None of the factors studied showed a significant differential association with uncontrolled hypertension.

**Table 5:** Factors associated with uncontrolled hypertension in treated patients and by sex.

	*Total population*			Female			Male		
	OR	95% CI	*P* value*	OR	95% CI	p	OR	95% CI	p
Increasing age	0.99	0.97-1.02	0.466	0.98	0.95-1.01	0.230	1.01	0.97-1.04	0.711
Male sex	1.05	0.83-1.33	0.582	….	….	….	….	….	…
Incresing level of education	1.01	0.82-1.26	0.901	1.07	0.82-1.39	0.614	0.89	0.62-1.30	0.553
Increasing wealth quintile	1.09	0.92-1.29	0.319	1.15	0.93-1.41	0.191	1.00	0.73-1.38	0.990
Diabetes mellitus	0.74	0.48-1.15	0.186	0.77	0.46-1.31	0.335	0.70	0.33-1.49	0.351
Hyperlipidaemia	1.02	0.67-1.55	0.944	0.98	0.59-1.63	0.942	1.16	0.52-2.58	0.721
Overweight/obesity**	0.67	0.37-1.22	0.186	0.77	0.35-1.68	0.509	0.48	0.17-1.37	0.167
Current smoking	1.14	0.44-2.95	0.786	1.84	0.07-49.8	…	1.20	0.44-3.29	0.721
Waist, cm	0.99	0.97-1.01	0.390	1.98	0.96-1.01	0.206	1.01	0.96-1.06	0.769
24-hour urinary salt excretion (mmol/L)	1.00	0.99-1.01	0.967	1.00	0.99-1.01	0.868	1.00	0.99-1.01	0.692

LR Logistic regression, OR odds ratio, CI confidence interval, BMI body mass index, *NM never married.* All calculations were done using complex samples – logistic regression. * Logistic regression, adjusted for increasing age, male sex, increasing level of education, wealth quintiles, presence of diabetes mellitus, hyperlipidaemia, overweight/obesity, current smoking, waist circumference and 24-hour urinary salt excretion in the model. ** BMI ≥ 23 kg/m^2^.

Weighted prevalences of undiagnosed hypertension among those who have hypertension in the population and in females and males were 1080(60.0%, weighted CI 58.1–63.4), 601 (53.6%, CI 50.1–57.0) and 479(69.4%, weighted CI 65.3–73.1) (p < 0.001), respectively. Risk associations for having undiagnosed hypertension are shown in Table 6. Undiagnosed hypertension in the population was positively associated with increasing age (p < 0.001), wealth quintile (p = 0.025), presence of other metabolic risk factors such as diabetes (p < 0.001) and hyperlipidaemia (p < 0.001), and high sodium intake (p = 0.046). However, it was negatively associated with current smoking (P = 0.017). The associations of undiagnosed hypertension in women were age (p = 0.03), presence of diabetes (p = 0.045) and hyperlipidaemia (p < 0.001). The same in males were age (p < 0.001), diabetes (p < 0.001), hyperlipidaemia (p < 0.001), overweight/obesity (P = 0.017), and current smoking (P = 0.015). However, Overweight/obesity was shown to have a significant association with undiagnosed hypertension only in males (p = 0.017) in stratified analyses.

**Table 6:** Factors associated with undiagnosed hypertension among those with hypertension and by sex.

	*Total population*			Female			Male		
	OR	95% CI	*P* value*	OR	95% CI	p	OR	95% CI	p
Increasing age	1.04	1.02-1.05	<0.001	1.02	1.01-1.04	0.003	1.06	1.03-1.08	<0.001
Male sex	1.05	0.83-1.33	0.145	….	….	….	….	….	…
Incresing level of education	0.91	0.81-1.02	0.106	0.87	0.75-1.02	0.087	0.95	0.78-1.17	0.636
Increasing wealth quintile	1.12	1.02-1.24	0.025	1.10	0.97-1.34	0.146	1.17	0.99-1.38	0.066
Diabetes mellitus	1.80	1.34-2.42	<0.001	1.85	1.30-2.64	<0.001	1.63	1.01-2.62	0.045
Hyperlipidaemia	3.81	2.80-5.19	<0.001	3.70	2.57-5.32	<0.001	3.98	2.33-6.80	<0.001
Overweight/obesity**	1.39	0.99-1.95	0.59	1.15	0.74-1.77	0.541	2.03	1.14-3.61	0.017
Current smoking	0.53	0.32-0.89	0.017	…	…	…	0.51	0.30-0.88	0.015
Waist, cm	1.00	0.99-1.02	0.078	1.01	0.99-1.02	0.330	1.00	0.96-1.01	0.372
24-hour urinary salt excretion (mmol/L)	1.00	0.99-1.00	0.046	1.00	0.99-1.002	0.189	0.99	0.99-1.00	0.067

LR Logistic regression, OR odds ratio, CI confidence interval, BMI body mass index. All calculations were done using complex samples – logistic regression. * Logistic regression, adjusted for increasing age, male sex, increasing level of education, wealth quintiles, presence of diabetes mellitus, hyperlipidaemia, overweight/obesity, current smoking, waist circumference and 24-hour urinary salt excretion in the model. ** BMI ≥ 23 kg/m^2^.

## Discussion

This national survey revealed that 37.2% of Sri Lankans, a South Asian population, had hypertension, and 60.0% of them were previously undiagnosed. Of the previously diagnosed patients with hypertension, the majority, 73.6%, were on treatment, but only 38.0% achieved BP targets. Undiagnosed hypertension was more common in men, with 69.4% being undiagnosed. However, the percentages of treated hypertension and of controlled on treatment did not differ between males and females. This highlights the need for screening and identifying undiagnosed patients with hypertension and targeting screening strategies, especially to detect males with undiagnosed hypertension, while concentrating on optimising BP control in those who are already on treatment. Further, we noted that the risk of hypertension was especially higher with metabolic risk factors like diabetes in women. At the same time, it was more strongly associated with lifestyle-related factors, such as high wealth quintile and sodium excretion in males. This highlights the importance of concentrating on differential preventive measures for females and males.

Our findings on the prevalence and control of hypertension in Sri Lanka largely agree with previous literature [7,8]. Prevalence of hypertension has increased from 28% in 2018–2019 to 37% in 2021, but the rates of untreated hypertension have not changed [7]. Our findings do keep in line with the world literature as well. According to the World Health Organization’s (WHO) first report on hypertension published in 2023, 30–35% globally have hypertension, with only 54% of adults being diagnosed, 42% receiving treatment, and a mere 21% having their hypertension controlled [19,20]. The prevalence of hypertension in Sri Lanka is comparable to that in India (30.7%), higher than in the United Kingdom (21.4%), but similar to the rate in the United States (31.7%) [21,22]. The rising prevalence of hypertension may be related to the growing elderly population and worsening lifestyle with urbanisation [23]. The disparity in prevalence of hypertension in Sri Lanka compared to the United Kingdom, but similarity with the United States, may be due to poor adherence to diet and lifestyle measures (high dietary fat and salt intake), low levels of physical activity, urbanisation, genetic predisposition and the deficits within the healthcare system [19,22]. Even though the undiagnosed hypertension in the total population of Sri Lanka is comparable to the global voluntary target of 21% and similar to Asian countries, except South Korea and Thailand [24], more than half of the patients with hypertension are undiagnosed and therefore it is a high-priority area to act on, as those people are at risk of hypertension-related complications, a significant proportion of preventable burden.

Gender related disparities in hypertension could arise related to different risk factors, which are significantly associated with hypertension in both genders, like age, education, alcohol consumption, wealth, diet intake, and obesity [25]. It is also reported that Sex differences in the prevalence of hypertension and uncontrolled hypertension vary by age [26]. Mechanisms responsible for these blood pressure differences may include the sustained vascular influence of hypertensive disorders of pregnancy, interactions between the renin-angiotensin-aldosterone system and sex hormones or even psychosocial gendered factors such as socioeconomic deprivation [27]. A study from Nigeria identified age, marital status, ethnicity, education and occupation as being significantly associated with hypertension among females. In contrast, age, marital status and ethnicity were significantly associated with elevated BP among male hypertensives [28]. Being female was associated with good blood pressure control in Germany [29]. However, we did not see a similar association in this South Asian population. There is evidence to suggest that women are partly at risk for hypertension and complications from hypertension due to inequalities in gender, domestic abuse, socioeconomic deprivation, poor health literacy, and environmental risk factors [30–34]. Prevalence of hypertension was higher in men than in women in France, and it was identified that socioeconomic status and education displayed a stronger association with hypertension prevalence in women compared to men [35]. However, our findings were slightly different from those of the West. Bangladesh reported a higher prevalence of hypertension in females, different to our findings [36]. In Kasakasthan, it is reported that Males exhibited higher cardiovascular risk behaviours and blood pressure. At the same time, females showed greater healthcare engagement and abdominal obesity, highlighting the need for gender-specific strategies in cardiovascular disease prevention and management [37].

Undiagnosed hypertension was more common among males in Sri Lanka. One main reason for this could be that more men are involved in economic activities and are not attending screening programs, as observed in our population (Table 1) and in previous literature [38]. Therefore, concrete, actionable screening recommendations, especially targeting males and the workforce, are needed. Further, as owerweight/obeseity was significantly more associated with undiagnosed hypertension, targeting all obese/overweight patients, especially males, is important. With the differential predilection observed with diabetes in females and high wealth quintile, and 24-hour urinary salt excretion in males, this points to addressing different prevention strategies in males and females. Therefore, the screening programs should also concentrate on wealthy men. Consideration of sex-related barriers to the screening program is important to address, especially in South Asians, as those economies are run by a male-dominated workforce with males working during the day, while a fair number of females are unemployed.

Sri Lanka has achieved a good treatment rate for diagnosed hypertensives, almost reaching the global target of 80%, which is higher than the global average of 42–44% and represents health equality in the country [19,39,40]. It is similar to the statistics for the United Kingdom and Canada, at 67–70%. This could be explained by Sri Lanka’s provision of free universal healthcare and the country’s high health literacy rate [19,39,40]. However, despite having a good treatment rate, the controlled BP rate was low at 38%. Even though this rate is better than other low-middle-income countries (LMIC), where the rate is around 20%, this is one area for improvement to reduce hypertension-related burden in the country to achieve at least the rates of the best centres in the world, such as in South Korea and Canada, where the control rate is around 57% ([19,41]. A study of ambulatory patients in the United States found that women were less likely than men to meet blood pressure control targets [42]. However, we did not identify a significant sex-related disparity in hypertension control rates in Sri Lanka. This may be because sex inequality in healthcare access is lower in Sri Lanka [38,39].

This study has several strengths. We used a nationally representative sample selected through a multistage, stratified cluster-random sampling design. The internationally validated STEPS questionnaire and protocol were used. Measurements were obtained in the participants’ own homes, potentially reducing the influence of factors such as hospital-induced anxiety or white coat hypertension. Blood pressure was measured using standardised digital automatic blood pressure meters to avoid clinician-based errors, and the mean of the last two BP values was used to avoid anxiety-related errors. Salt consumption was estimated using urinary sodium excretion values to avoid information bias. Finally, weighted analyses were performed according to the sampling method, and population adjustment for STEP1, STEP2, and STEP3 was performed according to WHO recommendations.

However, there are a few limitations to be acknowledged. Being a cross-sectional study, it limits the ability to infer causality, but allows for the presentation of associations with hypertension. The study sample showed a predominance of women, likely due to men's non-attendance at clinics, as they are often busy with their occupations. This could have led to some selection bias, but weighted analyses minimised this. A total of 6,267 patients were enrolled in step 1 of the STEPS survey, and only 4,634 attended step 2 for BP measurements. We analysed data of patients who attended BP step-2 of the survey. This could affect the results, as only the keen are likely to attend the clinics, and those not so keen on health may be missed. regularly. Blood pressure measurements were taken during a single visit to each household, rather than over multiple visits as recommended by current guidelines for diagnosing hypertension. This could have accounted for the result as a white-coat effect. However, we tried to minimise this limitation by taking three blood pressure readings during each visit and using the mean of the last two readings in the analyses.

## Conclusion

More than half of the hypertensives in this South Asian population were previously undiagnosed. Of hypertensives, ¾ were treated, but only 1/3 achieved treatment goals. Prevalence, treatment and control rates of hypertension were not different between females and males, but undiagnosed hypertension was common in men. Reducing inequalities in hypertension may require both gender-specific and general approaches. Therefore, the emphasis should be on improving the detection of hypertension, especially in men, and on treating blood pressure to targets in both sexes. This could be achieved by arranging compulsory workplace-based assessments, improving knowledge on hypertension and empowering patients to manage their blood pressure, and encouraging and educating treating physicians. The findings highlight the importance of conducting public awareness campaigns on hypertension, screening the public, especially targeting men and the workforce. Implementation of strategies that could help improve controlled BP rates in treated patients by updating physicians on the latest guidelines and educating the public on the importance of adherence to both non-pharmacological and pharmacological management is important. Alternative strategies, such as implementing protocol-based blood pressure management, can overcome physician inertia amid low patient-to-doctor ratios in LMICs such as Sri Lanka.
